# IGF-1-mediated PKM2/β-catenin/miR-152 regulatory circuit in breast cancer

**DOI:** 10.1038/s41598-017-15607-y

**Published:** 2017-11-21

**Authors:** Yi-Yang Wen, Wei-Tao Liu, Hao-Ran Sun, Xin Ge, Zhu-Mei Shi, Min Wang, Wei Li, Jian-Ying Zhang, Ling-Zhi Liu, Bing-Hua Jiang

**Affiliations:** 1 State Key Laboratory of Reproductive Medicine, Key Laboratory of Human Functional Genomics of Jiangsu Province, Collaborative Innovation Center for Cancer Personalized Medicine, Jiangsu Key Laboratory of Cancer Biomarkers, Prevention, and Treatment, Cancer Center, and Department of Pathology, Nanjing Medical University, Nanjing, Jiangsu China; 2 Department of Neurosurgery, The First Affiliated Hospital of Nanjing Medical University, Nanjing, China; 3 Department of Pathology, Affiliated Drum Tower Hospital of Nanjing University, Medical School, Nanjing, China; 4 Department of Biological Sciences & NIH-Sponsored Border Biomedical Research Center, The University of Texas at El Paso, El Paso, TX 79968 United States of America; 5 The Center for Molecular Carcinogenesis, Department of Pathology, Anatomy and Cell Biology, Thomas Jefferson University, Philadelphia, United States of America

## Abstract

Dysregulation of miRNAs is important in breast cancer initiation and malignant progression. Recently we showed that miR-152 downregulation is associated with breast cancer development, yet the underlying mechanism of miR-152 remains to be well elucidated. In this study, we identified β-catenin as a new direct target of miR-152. MiR-152 inhibited cell proliferation by targeting and inhibiting both β-catenin and PKM2 expression. We found that miR-152 expression sensitized the breast cancer cells to paclitaxel treatment by inhibiting β-catenin and PKM2 expression. Intriguingly, IGF-1 induced β-catenin and PKM2 expression and enhanced β-catenin and PKM2 interaction. Subsequently, IGF-1-induced β-catenin and PKM2 complex translocated into the nucleus, which in turn activated expression of miR-152. These results suggested a regulatory circuit between miR-152, β-catenin and PKM2 in breast cancer. By using human clinical specimens, we also showed that miR-152 expression levels were negatively correlated with β-catenin and PKM2 levels in breast cancer tissues. Our findings provide new insights into a mechanism of miR-152 involved in β-catenin and PKM2 inhibition which would have clinical implication for the cancer development and new treatment option in the future.

## Introduction

Breast cancer is the most frequent type of cancer in women^1^. Although the 5-year relative survival rate for female breast cancer patients has improved due to both improvements in breast cancer treatment and early detection, breast cancer is still the second leading cause of cancer deaths^2^. Therefore, further understanding of molecular mechanisms in breast cancer cells is important to develop new biomarkers and treatment options for breast cancer.

MiRNAs are a class of small endogenous non-coding RNAs composed of 17–24 nucleotides that act as post-transcriptional regulators through directly binding to the 3′-untranslated region (3′ UTR) of their target mRNAs, resulting in the degradation or translational inhibition of the mRNAs^3,4^. MicroRNA-152 (miR-152) contains two different mature miR-152 sequences, namely miR-152-5p and miR-152-3p. MiR-152-3p, the 3′ arm of the hairpin precursor, was highly conserved in evolution and has been well investigated in human cancers than miR-152-5p^5^. Recently, decreased expression of miR-152-3p (here referred as miR-152) has been observed in various types of human cancer cell lines and tumor tissues, such as ovarian^6^, gastrointestinal cancer^7^, hepatocellular carcinoma^8^, endometrial^9^ and breast cancer^10^. It was reported that the miR-152 directly targeted DNMT1 (DNA methyltransferase 1) in malignant cholangiocytes, leading to significant reduction of DNMT1 expression at both mRNA and protein levels^11^. This finding was further confirmed in subsequent studies on hepatitis B virus-related hepatocellular carcinoma^8^, ovarian cancer^6^, breast cancer^10^, pancreatic cancer^12^ and prostate cancer^13^. Except for DNMT1, accumulating evidence indicates that miR-152 targets on multiple oncogenes like PKM2, IRS-1 and IGF-1R in human breast cancer, and inhibits a variety of cellular functions, including proliferation, angiogenesis and migration, suggesting that miR-152 may potentially function as a tumor suppressor in breast cancer^8,10,14^.

β-catenin, the downstream molecule of IGF-1^15,16^, is originally identified as an important junctional component at the cell membrane, where it serves to link cadherin to the actin cytoskeleton via binding of α-catenin^17^. Alternatively, accumulation of β-catenin in the cytoplasm followed by its translocation and activation in the nucleus has also been well characterized as the central event in the progression of canonical WNT/β-catenin signaling^18^. In recent years, dysregulation of Wnt/β-catenin signaling pathway has been recognized as one of the hallmarks of breast cancer initiation and progression, mainly due to the abnormal excessive expression and the activating mutations of β-catenin^19,20^. Recent studies have illustrated the participation of miRNAs in the post-transcriptional regulation of WNT/β-catenin pathway, such as miRNA-720, miRNA-141 and miRNA-208a^21^. The objective of this study was to reveal the molecular mechanism of miR-152 and β-catenin in breast cancer. Previous study indicated that miR-152 directly targeted and inhibited PKM2 in breast cancer^14^. PKM2 is the key member of pyruvate kinase (PK) that catalyzes the final step in glycolysis by transferring the phosphate from phosphoenolpyruvate (PEP) to ADP, thereby generating pyruvate and ATP^22^. In recent years, numerous studies have indicated that PKM2 is preferentially expressed in malignant cancer, playing a vital role in cancer cell proliferation and tumor growth^23,24^.

The IGF signaling contains a dynamic network of proteins including ligands (insulin, IGF-1, IGF-2), their associated receptors (IGF-1R and IGF-2R) and several IGF binding proteins (IGFBPs) that participate in the regulation of human cancer development^25^. Of particular interest, IGF-1 has been most strongly implicated in breast cancer progression based on its mitogenic and anti-apoptotic activities^26^. Earlier studies have identified the association of IGF-1 with the increased risk of breast cancer development^27^. In addition, IGF-1 could bind with ER (estrogen receptor) or PR (progesterone receptor) to promote tumorigenesis and tumor growth in breast cancer^28,29^. Although multiple miRNAs, such as miR-122^30^, miR-18b^31^, miR-515-5p^32^, miR-148a and miR-152^10,14^, are involved in IGF-1 regulation pathway by directly targeting IGF-1, IGF-1R, IRS-1 or FOXO3a in breast cancer, the effects of IGF-1 induced signaling cascades on miRNA expression in breast cancer has yet to be evaluated.

In the present study, we showed that miR-152 was downregulated in breast cancer. We aim to address the following questions: (1) Whether β-catenin is direct target of miR-152 in breast cancer; (2) whether miR-152 overexpression inhibits cell proliferation by inhibiting both β-catenin and PKM2 expression; (3) what is role of miR-152 in breast cancer resistance to paclitaxel treatment; (4) whether miR-152 is involved in IGF-1-induced β-catenin and PKM2 expression. The answer of these questions would provide new insights into a better understanding of the role of miR-152 in breast cancer development.

## Results

### MiR-152 is downregulated in breast cancer tissues and cell lines

**Figure 1:**
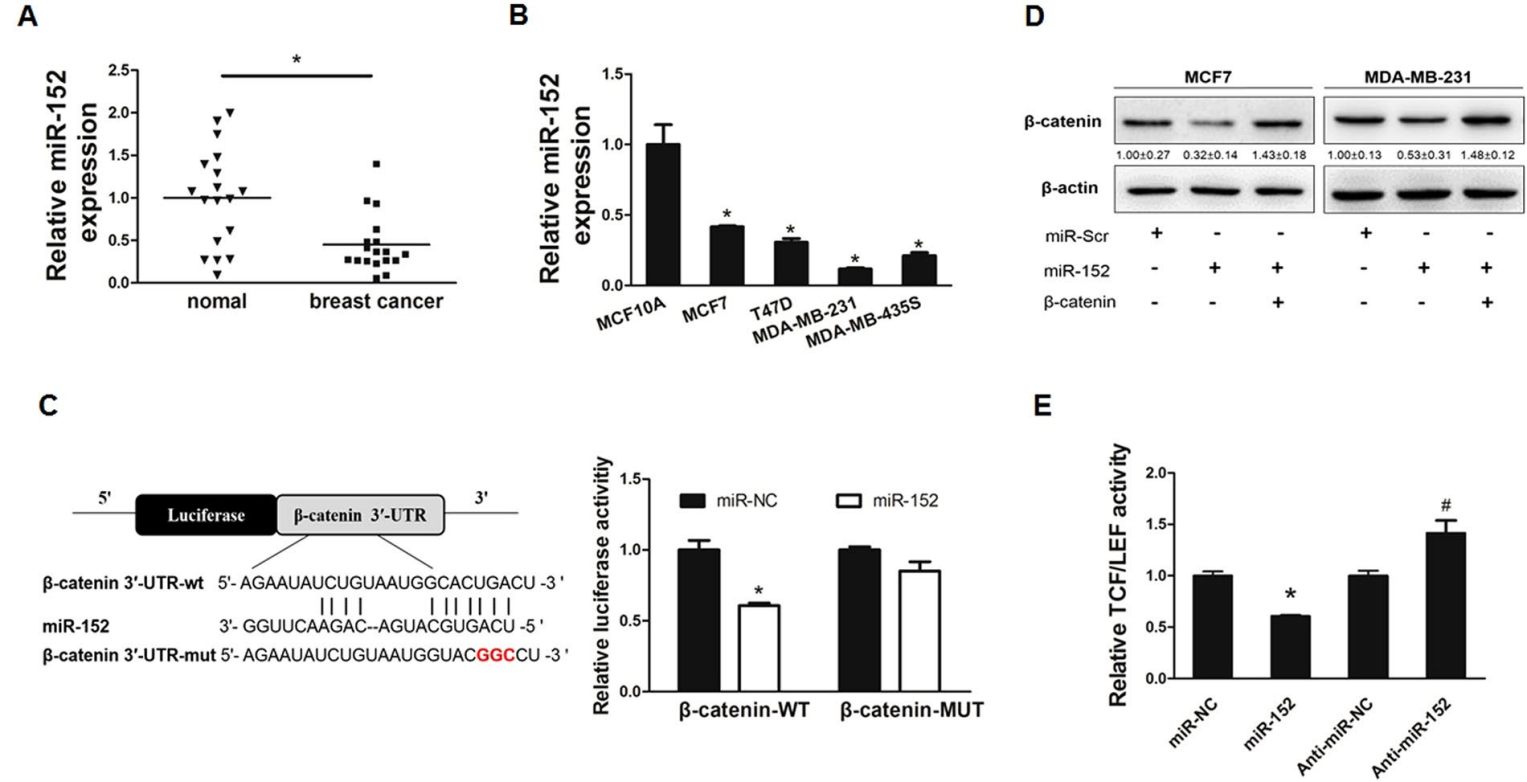
MiR-152 targeted β-catenin in breast cancer cells. **(A)** The relative expression levels of miR-152 (normalized to U6) in normal and breast cancer tissues were detected by qRT-PCR. **(B)** Expression levels of miR-152 in MCF-10A, MCF7, T47D, MDA-MB-453 and MDA-MB-231 cells were determined by qRT-PCR assay and normalized to the U6 levels. Results represent mean ± SEM from three independent experiments. *Indicates *p* < 0.05 when compared with miR-152 level in MCF-10A. **(C)** The sequence of miR-152 binding sites at 3′-UTR of β-catenin. The wild type (WT) and mutated (Mut) reporter constructs of the β-catenin 3′-UTR sequence are shown in left schematic diagram. Both constructs were verified by sequencing. Luciferase reporter assay was performed to detect the relative luciferase activities of WT and Mut β-catenin reporters, respectively. **(D)** Overexpression of miR-152 inhibited β-catenin expressions at protein level. Negative control (miR-NC) or miR-152 mimics (200 μM and 400 μM) were transiently transfected into MCF7 and MDA-MB-231 cells for 48 hours. Immunoblotting analysis was performed to detect the expression of β-catenin and β-actin. **(E)** MCF7 cells were co-transfected with TOP-FLASH/FOP-FLASH plasmids and negative control, miR-152 mimics or anti-miR-152. Luciferase activities were determined by the dual-luciferase reporter assay system. Data represent the means ± SEM of three independent experiments and * indicates *p* < 0.05 when compared with miR-NC group.

To test miR-152 expression levels in breast cancer tissues, quantitative real time PCR (qRT-PCR) analysis was performed to detect miR-152 expression levels in 18 pairs of breast cancer tissues and matched adjacent normal breast tissues. The results showed that the expression levels of miR-152 were significantly decreased in breast cancer tissues than those in noncancerous tissues (Fig. 1A). Similarly, the suppression of miR-152 expression also occurred in breast cancer cell lines MCF7, T47D, MDA-MB-231, MDA-MB-453 when compared with immortalized breast epithelial MCF-10A cells by qRT-PCR (Fig. 1B). Our results confirmed that miR-152 was downregulated in both breast cancer tissues and cancer cell lines.

### MiR-152 directly targets β-catenin in breast cancer cells

To further understand molecular mechanism of miR-152 in breast cancer cells, several well-developed miRNA algorithms, such as TargetScan, miRNApath, and TargetSearch, were employed to obtain a list of possible mRNA targets of miR-152. As a potential miR-152 binding site was predicted within the 3′-UTR of β-catenin mRNA, we constructed the 3′UTR reporters of β-catenin containing the miR-152 binding sites (WT) and corresponding mutant constructs (Mut) with 3 base pair substitution downstream of the pMIR-GLO luciferase reporters. HEK-293T (293 T) cells were co-transfected with miR-NC or miR-152 mimics, and reporter constructs, then the luciferase activity assay was performed after transfection for 48 hours. As shown in Fig. 1C, the luciferase activity of β-catenin WT 3′UTR was reduced by miR-152 overexpression, whereas the Mut constructs activity was not affected. In addition, to test whether miR-152 downregulates the expression of β-catenin, we transfected negative control (miR-NC) or miR-152 mimics into MCF7 and MDA-MB-231 cells, then detected β-catenin expression at the protein level. Immunoblotting results showed that miR-152 overexpression was sufficient to inhibit β-catenin protein expression (Fig. 1D). To further examine the reduction of β-catenin transactivation by miR-152, TCF/LEF-1 luciferase reporter analyses showed that force expression of miR-152 significantly inhibited β-catenin transactivation while downregulation of miR-152 by using anti-miR-152 promoted β-catenin transactivation (Fig. 1E). Thus, the suppression of β-catenin by miR-152 was functionally validated, suggesting that miR-152 specifically targeted β-catenin by binding its seed sequence to β-catenin 3′UTR region. Moreover, miR-152 significantly inhibited protein expression and transactivation of β-catenin.

### MiR-152 represses cell growth by both targeting β-catenin and PKM2

**Figure 2:**
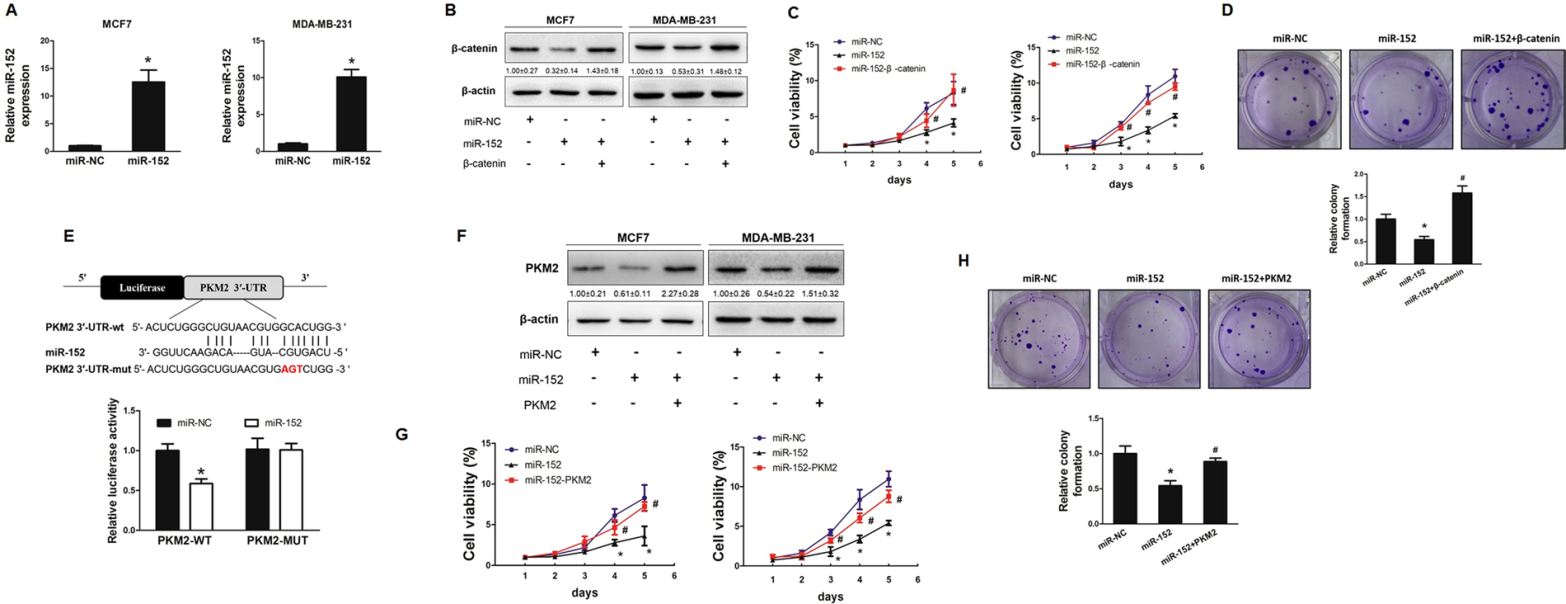
MiR-152 repressed cell growth via inhibiting both β-catenin and PKM2. **(A)** MCF7 and MDA-MB-231 cell lines stably expressing miR-152 or miR-NC were established by lentiviral transduction. The expression level of miR-152 (normalized to U6) in MCF7 and MDA-MB-231 cells stably overexpressing miR-152 or negative control of miRNA (miR-NC) were analyzed by qRT-PCR. *Indicates *p* < 0.05 compared with miR-NC group. **(B)** These miR-NC or miR-152 stably expressing cells were transfected with β-catenin cDNA plasmid without 3′-UTR. After transfection for 24 hours, cells were screened in 800 μg/ml G418 for a week. Total proteins were collected, then β-catenin and β-actin levels were determined by Immunoblotting assay. **(C)** CCK8 proliferation assay was performed every 24 hours (left: MCF7 cells; right: MDA-MB-231 cells). *p < 0.05. **(D)** Colony formation assay was performed using the stable MCF7 cells as described above. Cells were incubated for 14 days and the representative colonies were shown in the upper panel. **(E)** The wild type (WT) and mutated (Mut) reporter constructs of PKM2 3′-UTR sequences is shown. Relative luciferase activities of WT and Mut PKM2 reporters were detected by using Luciferase reporter assay. **(F)** PKM2 cDNA plasmid without 3′-UTR was transfected into miR-152 stably expressing cells as described above. Total proteins were collected and PKM2 levels were measured using Immunoblotting assay. **(G)** Cell viability was determined in MCF7 (left) and MDA-MB-231 (right) cells stably expressing miR-152 with or without PKM2 cDNA transfection, respectively. *Indicates *p* < 0.05 when compared to miR-NC group; ^#^indicates *p* < 0.05 when compared to miR-152 overexpression group. **(H)** Colony formation was tested using cells above. *Indicates *p* < 0.05 when compared to miR-NC group. ^#^Indicates *p* < 0.05 when compared to miR-152 overexpression group.

To assess whether miR-152 suppressed β-catenin expression by directly targeting its 3′UTR region, we established MCF7 and MDA-MB-231 cell lines stably expressing miR-152 or miR-NC using lentiviral transduction. The expression levels of miR-152 were analyzed in these stable cell lines using qRT-PCR (Fig. 2A). Then, the expression levels of β-catenin were determined by Immunoblotting. Cells overexpressed miR-152 showed low expression levels of β-catenin protein when compared with those of negative control cells. Moreover, miR-152-suppressed β-catenin expression was reversed by forced expression of β-catenin (Fig. 2B).

Given the important role of β-catenin in regulation of cell proliferation^22,33–35^, miR-152 overexpressed MCF7 and MDA-MB-231 cells were used to analyze cell proliferation. Cell proliferation was inhibited in miR-152-overexpressing MCF7 and MDA-MB-231 cells compared with miR-NC-overexpressing cells. Importantly, cell proliferation suppressed by miR-152 was rescued by overexpression of β-catenin cDNA without 3′UTR in MCF7 and MDA-MB-231 cells when compared with control group (Fig. 2C). Then, colony formation assay was performed to test whether β-catenin forced expression is sufficient to restore miR-152-inhibited cell transformation. Our results showed that forced expression of β-catenin restored miR-152-inhibited colony formation (Fig. 2D).

Our previous studies explored that miR-152 inhibited the Warburg effect by directly targeting PKM2^14^; while β-catenin had been reported to be an interacting protein of PKM2 in various cancer cells dependent on EGF stimulation. We predict that miR-152 simultaneously targets both PKM2 and β-catenin in breast cancer cells. Therefore, we performed studies to identify the known binding site of miR-152 in PKM2 3′UTR region. The luciferase activity of PKM2 3′UTR WT reporter, but not Mut reporter was significantly reduced in the cells transfected with miR-152 (Fig. 2E). Additionally, we observed that expression levels of PKM2 were downregulated in miR-152 stable expressing cells, and were restored in cells with forced expression of PKM2 without 3′UTR (Fig. 2F). Similarly, cell proliferation and colony formation were used to examine the involvement of PKM2 in miR-152-inhibited effects in the two different breast cancer cell lines. The results demonstrated that overexpression of PKM2 rescued miR-152-inhibited cell proliferation (Fig. 2G) and colony formation (Fig. 2H), confirming that PKM2 was also an important target of miR-152 for breast cancer cell proliferation and colony formation. Taken together, these data provide evidence that miR-152 may act as tumor suppressor in breast cancer cells by inhibiting cell growth and colony formation via inhibiting both β-catenin and PKM2.

### MiR-152 sensitizes the breast cancer cells to paclitaxel treatment by decreasing β-catenin and PKM2 expression

**Figure 3:**
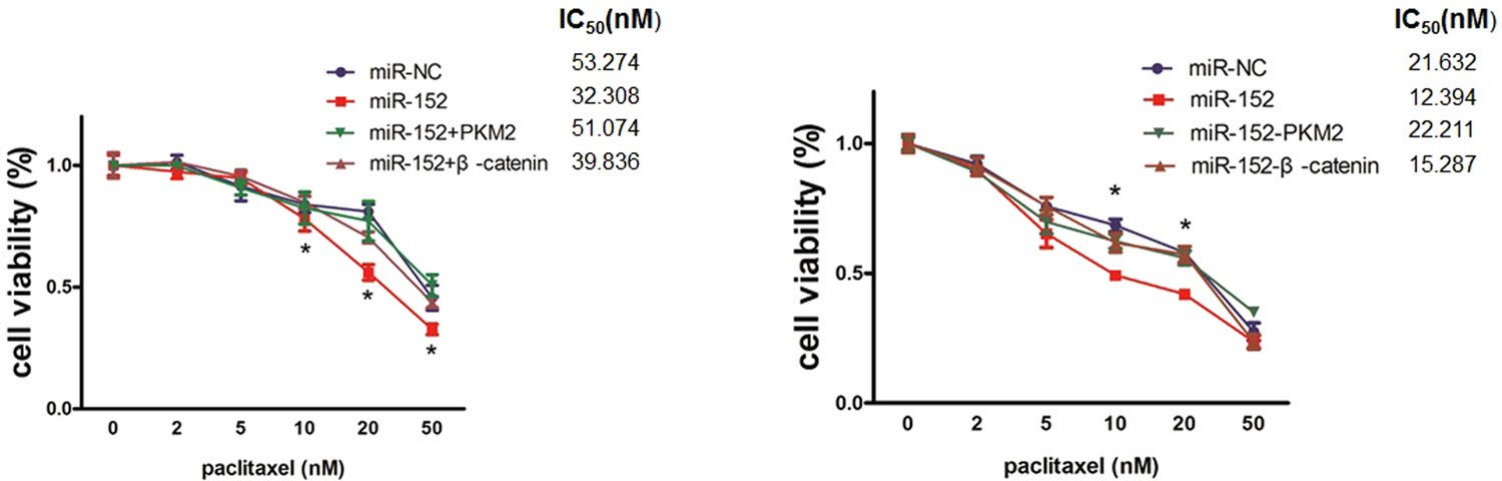
MiR-152 sensitized the breast cancer cells to paclitaxel treatment by β-catenin and PKM2 repression. MCF7 and MDA-MB-231 cells stably overexpressed miR-152 were transfected with β-catenin or PKM2 cDNA plasmid. Cells were counted and exposed to various concentrations (0, 2 nM, 5 nM, 10 nM, 20 nM, 50 nM) of paclitaxel for 48 hours. IC_50_ values were determined by CCK8 assay, (left: MCF7 cells; right: MDA-MB-231 cells). *Indicates *p* < 0.05 when compared to miR-NC control group.

Aberrant miRNA expression has been implicated to affect the response to various chemotherapy in breast cancer. Paclitaxel remains a major drug for breast cancer treatment, especially for recurrent or metastatic breast cancer^36^. Based on the potential roles of β-catenin and PKM2 in paclitaxel resistance, we conducted CCK8 assay to measure cell viability of MCF7 and MDA-MB-231 stable cells treated with different concentrations of paclitaxel. It was observed that overexpression of miR-152 significantly sensitized the breast cancer cells to paclitaxel treatment; however, the chemotherapy sensitivity due to miR-152 overexpression was significantly reversed by overexpression of PKM2. Additionally, overexpression of β-catenin in these cells also partially reversed the paclitaxel sensitivity, demonstrating that both of PKM2 and β-catenin are involved in miR-152-induced paclitaxel treatment response and PKM2 played a more sensitive role in paclitaxel resistance of breast cancer (Fig. 3).

### IGF-1 induces β-catenin and PKM2 expression and nuclear accumulation

**Figure 4:**
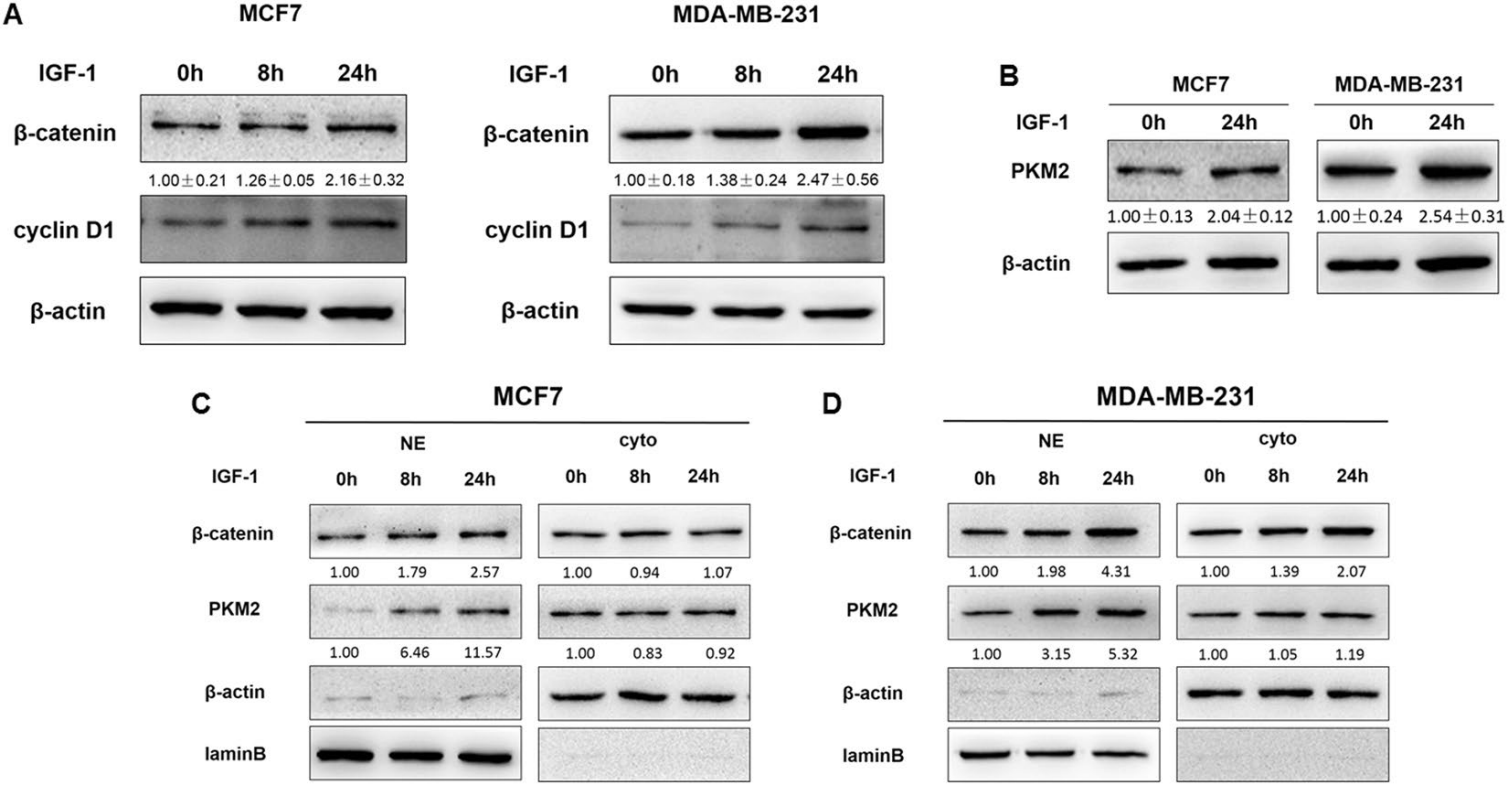
IGF-1 induced β-catenin and PKM2 expression and nuclear accumulation. **(A)** MCF7 and MDA-MB-231 cells were treated with 100 nM IGF-1 for 0, 8 and 24 hours, and the protein expression levels of β-catenin, cyclin D1, and β-actin were analyzed by Immunoblotting assay. **(B)** PKM2 expression levels in MCF7 and MDA-MB-231 cells treated with 100 nM IGF-1 for 24 hours were analyzed by Immunoblotting assay. **(C,D)** Immunoblotting assay of β-catenin and PKM2 levels in cytoplasmic extracts (Cyto) and in nuclear extracts (NE) in MCF7 and MDA-MB-231 cells treated with 100 nM IGF-1 for 0, 8 and 24 hours.

Earlier study has shown that EGF stimulation induced PKM2–β-catenin interaction and translocation into the nucleus in multiple types of cancer cells^37^. Given the important role of IGF-1 system in breast cancer cells and the ensure response of both MCF7 and MDA-MB-231 cells to IGF-1, we treated these cells with IGF-1 for 0, 8, and 24 hours. As shown in Fig. 4A, protein expression levels of β-catenin and cyclin D1 were increased after IGF-1 treatment. In addition, IGF-1 exposure for 24 hours also caused the significant induction of total PKM2 expression levels in both MCF7 and MDA-MB-231 cells (Fig. 4B). Notably, accumulation of β-catenin often results in β-catenin nuclear translocation and downstream gene expression. To examine whether IGF-1 specifically induces nuclear translocation of β-catenin and PKM2, subcellular distributions of β-catenin and PKM2 were measured by Immunofluorescence analysis. The data showed that IGF-1 incubation for 24 hours significantly increased the nuclear accumulation of β-catenin and PKM2 in both MCF7 and MDA-MB-231 cell lines (Supplementary Figure S1). Moreover, cell fractionation analysis was also used to assess the subcellular localization of β-catenin and PKM2 in MCF7 and MDA-MB-231 cells with IGF-1 treatment for 24 hours (Fig. 4 C and D). These results indicated that IGF-1 induced the overexpression of β-catenin and PKM2 and promoted their nuclear translocation in breast cancer cells.

### IGF-1 enhanced the binding of β-catenin and PKM2

**Figure 5:**
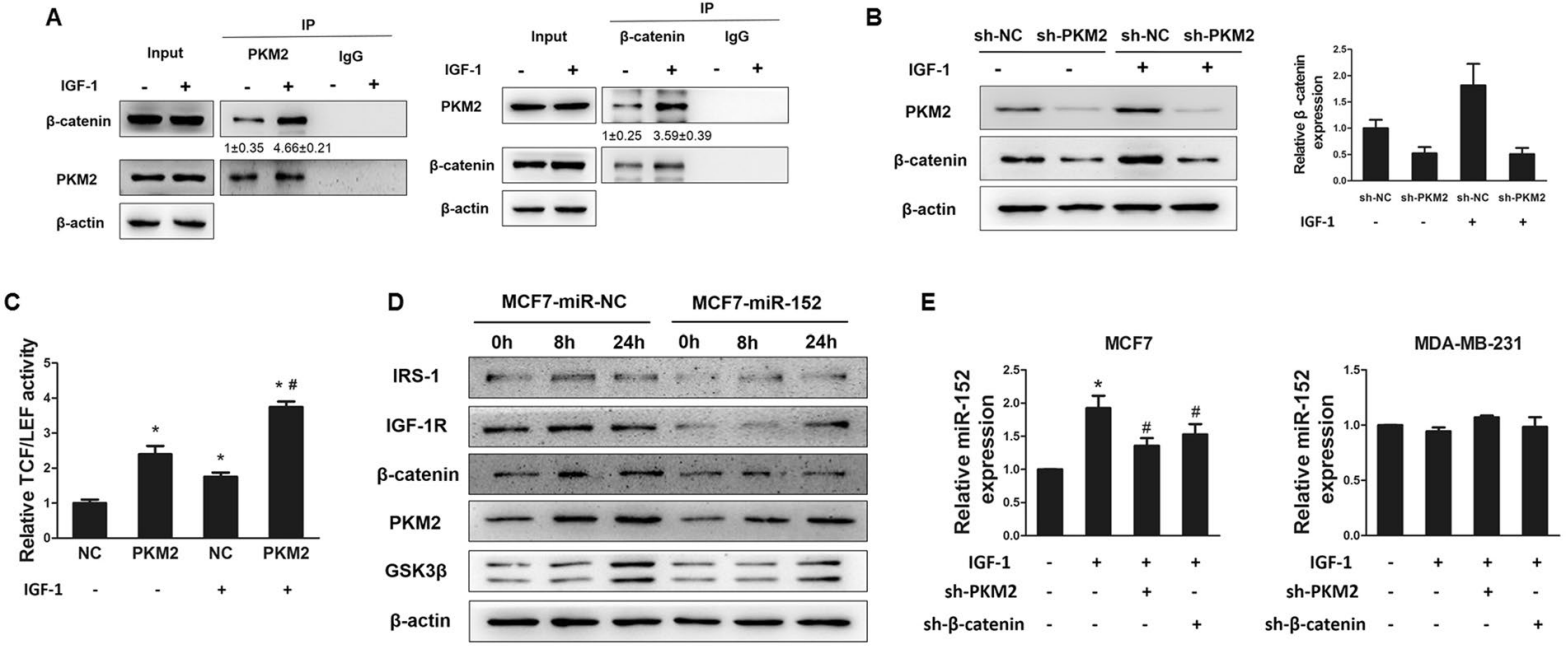
IGF-1 enhanced the combination of β-catenin and PKM2. **(A)** Immunoprecipitation (Co-IP) was performed on 293 T cells transfected with β-catenin and PKM2. After transfection for 48 hours, cells were subjected to Co-IP analysis using the indicated β-catenin and PKM2 antibodies for IP and blotting, with 10% of input proteins as indicated. **(B)** MCF7 cells were infected with lentivirus carrying sh-NC or sh-PKM2 plasmid and selected using puromycin. Then the cells were treated with IGF-1 for 24 hous after depletion of PKM2. Protein expression levels of PKM2 and β-catenin were analyzed by Immunoblotting assay (left), and the expression level of β-catenin was normalized to β-actin (right). The results were from three different replicates. **(C)** The stable PKM2-overexpressing MCF7 cells were transfected with TOP-FLASH/ FOP-FLASH plasmids and subsequently treated with IGF-1 for 24 hours. Luciferase activities were determined by the dual-luciferase reporter assay system. *Indicates *p* < 0.05 when compared to negative control (NC, without PKM2 overexpression) without IGF-1 treatment. ^#^indicates *p* < 0.05 when compared to NC with IGF-1 treatment. **(D)** MCF7 cells stably expressed miR-NC or miR-152 were treated with 100 nM IGF-1 for 0, 8 and 24 hours. The levels of indicated proteins were analyzed by Immunoblotting. **(E)** MiR-152 expression levels in MCF7 and MDA-MB-231 cells overexpressing sh-RNA negative control or sh-PKM2 or sh-β-catenin in combination with IGF-1 treatment for 24 hours were analyzed using qRT-PCR. *Indicates *p* < 0.05 when compared to negative control without any treatment; ^#^indicates *p* < 0.05 when compared to IGF-1 treatment group.

Above results showed that IGF-1 increased expression levels of PKM2 and β-catenin. We performed co-immunoprecipitation (Co-IP) analysis to further explore the interplay between β-catenin and PKM2 in response to IGF-1 stimulation. Our results showed a stronger interaction between β-catenin and PKM2 by IGF-1 treatment compared with the solvent (Fig. 5A). These results suggested that IGF-1 enhanced the expression levels of β-catenin and PKM2 and promoted their interaction, possibly leading to their nuclear translocation. In agreement with our earlier data, we hypothesize that IGF-1-enhanced binding of β-catenin and PKM2 may be responsible for β-catenin and PKM2 overexpression. To further dissect whether the IGF-1 dependent effects on β-catenin in MCF7 cells were mediated by PKM2, we performed Immunoblotting analysis to detect IGF-1-stimulated protein levels of β-catenin with PKM2 knockdown in MCF7 cells. MCF7 cells were transfected with lentivirus carrying sh-PKM2 and selected by puromycin to construct PKM2 knockdown cells. The Immunoblotting analysis results showed that PKM2 depletion significantly blocked IGF-1-induced β-catenin expression, suggesting that IGF-1-triggered expression of PKM2 could play an important role in IGF-1 associated β-catenin enrichment (Fig. 5B). In addition, TCF/LEF-1 luciferase reporter analyses also indicated the overexpression of PKM2 remarkably enhanced IGF-caused β-catenin transactivation (Fig. 5C). Overexpression of miR-152 significantly repressed PKM2, β-catenin, IGF-1R and IRS-1 expressions, but not GSK3β, which was not a target of miR-152 (Fig. 5D). In addition, IGF-1 incubation significantly induced expression levels of miR-152 in MCF7 cells, but not in MDA-MB-231 cells; while depletion of PKM2 or β-catenin partly abrogated the IGF-1-induced miR-152 upregulation in MCF7 cells, but not in MDA-MB-231 cells (Fig. 5E). We also interfered IGF-IR in breast cancer cell lines and the results showed that miR-152 expression level was inhibited by IGF-1R knockdown (Supplementary Figure S2).

### Correlation of miR-152 suppression, β-catenin and PKM2 overexpression in clinical breast cancer tissues

**Figure 6:**
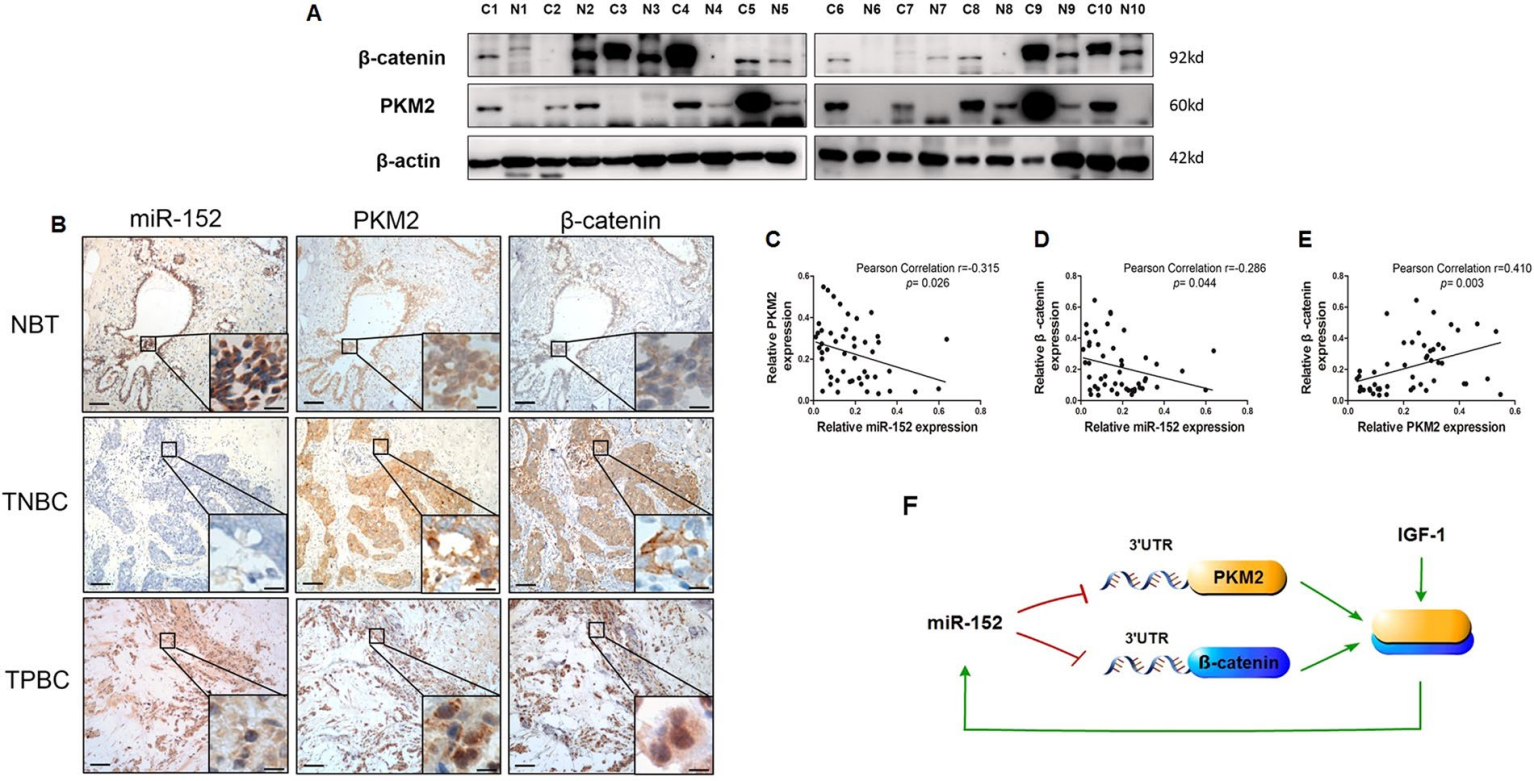
The miR-152 expression levels was negatively correlated to β-catenin and PKM2 levels in breast cancer patients. **(A)** Representative of Immunoblotting images showing expression levels of β-catenin and PKM2 in 10 pairs of adjacent normal breast tissues (N) and breast tumor tissues (**C**). The expression of β-actin was used as an internal control. **(B)** Immunohistochemical (IHC) staining of β-catenin and PKM2 and *in situ* hybridization (ISH) staining of miR-152 in representative normal breast tissues (NBT), triple negative breast cancer (TNBC) and triple positive breast cancer (TPBC) specimens (×200 and ×400 magnification; Scale bar: left panel, 50 μm; right panel, 20 μm). **(C,D)** Pearson correlation analysis was used to determine the corrections between the β-catenin/PKM2 expression and miR-152 levels in human breast cancer specimens. **(E)** The correlation between the β-catenin and PKM2 expression levels was performed by pearson correlation analysis. The data was collected and quantified by Image-pro plus 6.0 from IHC and ISH staining in human breast cancer specimens. **(F)** In breast cancer cells, miR-152 directly targets and inhibits both PKM2 and β-catenin. Furthermore, IGF-1 induces PKM2 and β-catenin expression and enhances the interaction of PKM2 and β-catenin. Subsequently, IGF-1-induced PKM2 and β-catenin complex translocates into nucleus, which in turn activates the expression of miR-152.

β-catenin and PKM2 expression levels in 10 pairs of breast cancer tissues were detected by Immunoblotting. Consistent with previous results in breast cancer cells, protein levels of β-catenin and PKM2 were much higher in most cases of cancer tissues than those in their matched adjacent normal tissues. The representative images was shown in Fig. 6A. Next, we examined β-catenin, PKM2 and miR-152 expression levels in 25 pairs of breast cancer specimens and their matched adjacent normal breast tissues (NBT), including 9 triple negative breast cancer (TNBC) tissues and 16 triple positive breast cancer (TPBC) tissues by using *In situ* hybridization and Immunohistochemical staining (Fig. 6B). Overexpression of β-catenin and PKM2 was observed both in TNBC and TPBC tissues, but not in NBT tissues, while miR-152 levels were much higher in NBT, and lower or non-expressed in TPBC and TNBC tissues, confirming that expression levels of miR-152 were inversely correlated with the β-catenin and PKM2 expressions levels in these tissues (Table 1). Pearson Correlation analysis showed the significant negative correlations between miR-152 and PKM2 (correlation = 0.−315, *p* = 0.026), and β-catenin (correlation = 0.−286, *p* = 0.044), respectively. Similarly, the correlation of PKM2 with β-catenin (correlation = 0.410, *p* = 0.003) was also significantly different (Fig. 5 C,D,E). These results indicated the highly negative correlations between expression levels of miR-152 and its target proteins: β-catenin and PKM2 in human breast cancer tissues.

**Table 1:** The expression of miR-152, PKM2 and β-catenin in breast cancer patients.

total	Breast cancer			Normal breast tissues		
	n = 25			n = 25		
	miR-152	PKM2	β-catenin	miR-152	PKM2	β-catenin
**−**	12	2	3	2	10	13
**+**	6	2	3	8	6	8
**++**	4	10	6	8	6	3
**+++**	3	11	13	7	3	1

“−” means negative staining; the positive staining was gradually increased from “**+**” to “**+++**”.

## Discussion

Recent studies have indicated the involvement of different miRNAs in breast cancer proliferation, differentiation, apoptosis and chemotherapy by regulating the post-transcriptional expression of mRNAs^38,39^. MiR-152 downregulation is associated with breast cancer development. Overexpression of miR-152 significantly inhibited cell proliferation, colony formation and tumor angiogenesis by targeting IGF-1R and IRS1 in breast cancer. Furthermore, DNA methyltransferase 1 (DNMT1), a target of miR-152, mediated CpG island hypermethylation of miR-152 gene promoter that may suppress miR-152 expression as a feedback regulatory mechanism^10^. In addition, miR-152 plays an important role in breast cancer cell proliferation and angiogenesis via PKM2/NF-κB/EGR1/miR-152 complex in respond to IGF-1R activation^14^. In this study, our results identified β-catenin as a new target of miR-152.

Recently, increasing evidence showed that miRNAs can affect sensitivity of breast cancer to paclitaxel treatment. For example, miR-101 could increases paclitaxel sensitivity by suppressing MCL-1 expression in human triple-negative breast cancer^40^. MiR-520h is involved in paclitaxel chemosensitivity by modulating DAPK2 expression^41^. Both β-catenin and PKM2 were implicated to play the important roles in paclitaxel resistance of breast cancer^42–44^. Our data showed that miR-152 sensitized breast cancer cells to paclitaxel treatment by targeting β-catenin and PKM2 repression, suggesting a novel role of miR-152 in breast cancer paclitaxel resistance. Thus, the combination treatment of miR-152 with paclitaxel could provide a new strategy to overcome paclitaxel resistance in breast cancer in the future.

Although IGF-1 has been well demonstrated to enhance breast cancer cell proliferation, only a small subset of miRNAs are shown to be significantly regulated by IGF-1 treatment in breast cancer^45^. Recent study has suggested that IGF-1 induced PKM2 phosphorylation, thus resulting in PKM2 overexpression and nuclear translocation^46^. Our results showed that IGF-1 significantly increased the expression levels of β-catenin and PKM2, enhanced their interaction, and promoted their nuclear accumulation, thus leading to miR-152 transcriptional activation. Moreover, downregulation of PKM2 or β-catenin abrogated the IGF-1-induced miR-152 expression, suggesting that miR-152 was involved in a new IGF-1-mediated miR-152/PKM2/β-catenin regulatory circuit in breast cancer. However, IGF-1-induced upregulation of miR-152 expression was not observed in MDA-MB-231 cells. It is possible that the hypermethylation of CpG islands in miR-152 gene promoters were found in MDA-MB-231 cells, which may abrogate the binding of many transcription factors, such as EGR1^14^. Additionally, the lower miR-152 expression level in MDA-MB-231 cells may be related to the hypermethylation of miR-152 gene promoter.

Taken together, our results reveal the vital role of miR-152 as a tumor suppressor in human breast cancer by inhibiting both β-catenin and PKM2 expression levels at the post-transcriptional level, and point out the potential clinical application of miRNA-analog for the cancer treatment.

## Materials and Methods

### Cell culture and regents

Human breast cancer cells MCF7, MDA-MB-231, T47D, MDA-MB-453, and human mammary epithelial cell line MCF10A were obtained from American Type Culture Collection. All breast cancer cells were maintained in high glucose DMEM (Invitrogen) supplemented with 10% fetal bovine serum, 100 units/mL penicillin and 100 μg/mL streptomycin. MCF-10A was cultured in DMEM/Ham’s F-12 medium supplemented with growth factors and 100 ng/ml Cholera Toxin. All cells were incubated in a 5% CO_2_ at 37 °C. β-catenin antibodies were from Cell signaling technology (CST, Beverly, MA, USA); β-actin antibody were from sigma (St. Louis, MO, USA); GSK3β and PKM2 antibodies were from Signalway Antibody (SAB, College Park, MD, USA); laminB antibodies were from Epitomics (Burlingame, CA, USA). The IGF-1 cytokine was purchased from sigma.

### Tissue samples

Tissues and pathological slides of breast cancer and matched adjacent normal breast tissues were obtained from our tissue bank in Nanjing medical university. The tissue samples were collected immediately after the surgical removal, and snap-frozen in liquid nitrogen. The specimens had been fixed in formalin and embedded in paraffin before they were archived. None of the patients received preoperative chemotherapy.

### Ethics statements

All experimental methods were performed in accordance with the relevant guidelines and regulations. For the use of clinical materials, written informed consent was obtained from all patients for research purposes, and the study was approved by the Institutional Research Ethics Committee of Nanjing medical university.

### Establishment of stable cell lines

The miR-152-overexpressing MCF7 and MDA-MB-231 cell lines were constructed by using a lentiviral packaging kit. Lentivirus carrying hsa-miR-152 or hsa-miR-NC was packaged following the manufacturer’s manual. Then these miR-NC or miR-152 stably expressing cells were transfected with β-catenin and PKM2 cDNA constructs without 3′UTR region. After transfection for 24 hours, cells were selected in culture with 800 μg/ml G418 for a week.

### Colony formation assay

MCF7 cells were seeded into the 24 well plate for about 3000 per well, and cultured for 2 weeks. All colonies were stained with 50% methanol and 0.5% crystal violet mixture. Colonies with diameter more than 1.0 mm were counted. The experiments were performed with three replicates, and repeated for three times.

### Cell proliferation assay

Cells were trypsinized, counted and plated in 96-well plates for about one thousands cells per well. The proliferation of the cells was measured using a Cell Counting Kit-8 (CCK-8) (Dojindo Laboratories, Kumamoto, Japan) according to the manufacturer’s instruction. All results were obtained from three separate experiments with six replicates per experiment.

### Protein extraction and Immunoblotting

Total proteins were extracted from MCF7 and MDA-MB-231 cells using RIPA lysis buffer with protease inhibitors (Protease Inhibitor Cocktail, 1 mM sodium orthovanadate, 2 mM DTT, 2 mM leupeptin). Aliquots of 20 ul lysates per sample were electro-transferred on 10% SDS-PAGE gel, following by transfer to PVDF membranes. Membranes were blocked with 5% skimmed milk and incubated with the appropriate antibodies.

### Isolation of total RNA and qRT-PCR analysis

Total RNAs were extracted using Trizol (Invitrogen) according to manufacturer’s instructions, after cells were washed by ice-cold PBS and scraped from dishes. The expression level of miR-152, PKM2 and β-catenin in breast cancer cells and tissues were measured by quantitative real time RT-PCR (qRT-PCR). All the primers used in this study were shown in Supplementary Table S1. qRT-PCR was carried out by the 7900HT Real-Time PCR Detection System (R&B) with Real-time PCR Master Mix (SYBR Green).

### *In situ* hybridization

Slides were treated and hybridized with 10 pmol probe (LNA-modified and DIG labeled oligonucleotide) complementary to miR-152, according to the manufacturer’s instructions. After incubation with anti-DIG-HRP Fab fragments conjugated to horseradish peroxidase, the hybridized probes were detected by incubating with 3,3′-diaminobenzidine (DAB) solution, and nuclei were counterstained with Carazzi’s Haematoxylin. The assessment of signals was performed by two experienced pathologists in a blind manner.

### Immunoprecipitation (Co-IP)

Proteins from 293 T cells transfected with PKM2 and β-catenin were extracted by RIPA lysis buffer. The protein extraction was incubated with indicated antibody (5–10 μg) for 16 hours at 4 °C followed by incubation with protein A/G agarose. The beads were washed five times with 1 ml of IP buffer. The samples were detected using Immunoblotting with indicated antibodies.

### Luciferase Reporter Assay

The 3′-UTR regions of β-catenin and PKM2 containing predicted miR-152 seed-matching sites (wide type, WT; mutant type, Mut) were inserted into pMIR-GLO vector (Ambion, CA, USA). 293 T cells were seeded into a 24-well plate for luciferase assay. After cultured overnight, cells were cotransfected with the WT or Mut plasmid and equal amounts of miR-152 or miR-NC using Lipofectamine 2000 (Invitrogen) according to the manufacturer’s instruction. Luciferase assays were performed 24 hours after transfection using the Dual Luciferase Reporter Assay System (Promega, WI, USA).

### Immunohistochemistry assay

For Immunohistochemistry (IHC), 4 μm sections of formalin-fixed and paraffin breast cancer tissues were divided into three groups (NBT, TNBT and TPBC) according to clinical diagnoses. After incubation with hydrogen peroxide, the sections were blocked for 1 hours with 10% goat serum and incubated with the indicated antibodies at 4 °C for 16 hours. After washing, the slides were incubated with HRP-conjugated secondary antibodies for 40 minutes (Proteintech). The antibody signals were detected using DAB regent. Sections incubated with the preimmune IgG were used as the negative control. Stained sections were examined under a light microscope (Zeiss).

### Immunofluorescence assay

MCF7 and MDA-MB-231 cells were incubated with or without IGF-1. After incubation for 24 hours, Cells were fixed with 4% formaldehyde at 4 °C, and blocked with 1% bovine serum albumin (BSA) in PBST (PBS containing 0.05% Tween-20). Cells were immunostained with antibodies against PKM2 or β-catenin overnight. The antigen-primary antibody complex was detected using fluorescence isothiocyanate (FITC) secondary antibodies (Proteintech). Microscopic observation was performed under a fluorescence microscope (OLYMPUS IMAGING).

### Statistical analysis

The data were analyzed by SPSS 16.0 and presented as the mean ± SEM. Student’s test and ANOVA were performed to compare the differences. Pearson’s correlation analysis was used to calculate the correlation between two groups. Differences were considered to be statistically significant at *p* < 0.05.

## Electronic supplementary material

Supplemental materials

## References

[1] Ferlay J (2015). Cancer incidence and mortality worldwide: sources, methods and major patterns in GLOBOCAN 2012. International journal of cancer.

[2] Xue J (2016). MiRNA-621 sensitizes breast cancer to chemotherapy by suppressing FBXO11 and enhancing p53 activity. Oncogene.

[3] Lee RC, Ambros V (2001). An extensive class of small RNAs in Caenorhabditis elegans. Science.

[4] Ambros V (2001). microRNAs: tiny regulators with great potential. Cell.

[5] Liu X, Li J, Qin F, Dai S (2016). miR-152 as a tumor suppressor microRNA: Target recognition and regulation in cancer. Oncology letters.

[6] Xiang Y (2014). MiR-152 and miR-185 co-contribute to ovarian cancer cells cisplatin sensitivity by targeting DNMT1 directly: a novel epigenetic therapy independent of decitabine. Oncogene.

[7] Chen Y (2010). Altered expression of MiR-148a and MiR-152 in gastrointestinal cancers and its clinical significance. Journal of gastrointestinal surgery: official journal of the Society for Surgery of the Alimentary Tract.

[8] Huang J, Wang Y, Guo Y, Sun S (2010). Down-regulated microRNA-152 induces aberrant DNA methylation in hepatitis B virus-related hepatocellular carcinoma by targeting DNA methyltransferase 1. Hepatology.

[9] Tsuruta T (2011). miR-152 is a tumor suppressor microRNA that is silenced by DNA hypermethylation in endometrial cancer. Cancer research.

[10] Xu Q (2013). A regulatory circuit of miR-148a/152 and DNMT1 in modulating cell transformation and tumor angiogenesis through IGF-1R and IRS1. Journal of molecular cell biology.

[11] Braconi C, Huang N, Patel T (2010). MicroRNA-dependent regulation of DNA methyltransferase-1 and tumor suppressor gene expression by interleukin-6 in human malignant cholangiocytes. Hepatology.

[12] Azizi M (2014). *MicroRNA-148b and microRNA-*152 reactivate tumor suppressor genes through suppression of DNA methyltransferase-1 gene in pancreatic cancer cell lines. Cancer biology & therapy.

[13] Zhu C (2013). miR-152 controls migration and invasive potential by targeting TGFalpha in prostate cancer cell lines. The Prostate.

[14] Xu Q (2015). Regulatory circuit of PKM2/NF-kappaB/miR-148a/152-modulated tumor angiogenesis and cancer progression. Oncogene.

[15] Chen J (2005). Functional significance of type 1 insulin-like growth factor-mediated nuclear translocation of the insulin receptor substrate-1 and beta-catenin. The Journal of biological chemistry.

[16] Sunters A (2010). Mechano-transduction in osteoblastic cells involves strain-regulated estrogen receptor alpha-mediated control of insulin-like growth factor (IGF) I receptor sensitivity to Ambient IGF, leading to phosphatidylinositol 3-kinase/AKT-dependent Wnt/LRP5 receptor-independent activation of beta-catenin signaling. The Journal of biological chemistry.

[17] Kemler R (1993). From cadherins to catenins: cytoplasmic protein interactions and regulation of cell adhesion. Trends in genetics: TIG.

[18] Clevers H, Nusse R (2012). Wnt/beta-catenin signaling and disease. Cell.

[19] Lin SY (2000). Beta-catenin, a novel prognostic marker for breast cancer: its roles in cyclin D1 expression and cancer progression. Proceedings of the National Academy of Sciences of the United States of America.

[20] Khramtsov AI (2010). Wnt/beta-catenin pathway activation is enriched in basal-like breast cancers and predicts poor outcome. The American journal of pathology.

[21] Huang K (2010). MicroRNA roles in beta-catenin pathway. Molecular cancer.

[22] Israelsen WJ (2013). PKM2 isoform-specific deletion reveals a differential requirement for pyruvate kinase in tumor cells. Cell.

[23] Dong G (2016). PKM2 and cancer: The function of PKM2 beyond glycolysis. Oncology letters.

[24] Canal F, Perret C (2012). PKM2: a new player in the beta-catenin game. Future oncology.

[25] Nieto-Estevez V, Defterali C, Vicario-Abejon C (2016). IGF-1: A Key Growth Factor that Regulates Neurogenesis and Synaptogenesis from Embryonic to Adult Stages of theBrain. Frontiers in neuroscience.

[26] Cleveland RJ (2006). IGF1 CA repeat polymorphisms, lifestyle factors and breast cancer risk in the Long Island Breast Cancer Study Project. Carcinogenesis.

[27] Hankinson SE (1998). Circulating concentrations of insulin-like growth factor-I and risk of breast cancer. Lancet.

[28] Lee AV, Yee D (1995). Insulin-like growth factors and breast cancer. Biomedicine & pharmacotherapy = Biomedecine & pharmacotherapie.

[29] Yee D, Lee AV (2000). Crosstalk between the insulin-like growth factors and estrogens in breast cancer. Journal of mammary gland biology and neoplasia.

[30] Wang B, Wang H, Yang Z (2012). MiR-122 inhibits cell proliferation and tumorigenesis of breast cancer by targeting IGF1R. PloS one.

[31] Wu JH (2016). *MiR-18b suppresses high-glucose-induced proliferation in HR*ECs by targeting IGF-1/IGF1R signaling pathways. The international journal of biochemistry & cell biology.

[32] Gilam A (2013). Involvement of IGF-1R regulation by miR-515-5p modifies breast cancer risk among BRCA1 carriers. Breast cancer research and treatment.

[33] Harris I, McCracken S, Mak TW (2012). PKM2: a gatekeeper between growth and survival. Cell research.

[34] Xu J (2016). beta-catenin regulates c-Myc and CDKN1A expression in breast cancer cells. Molecular carcinogenesis.

[35] Hsieh TH (2016). A novel cell-penetrating peptide suppresses breast tumorigenesis by inhibiting beta-catenin/LEF-1 signaling. Scientific reports.

[36] Cardoso F (2011). Locally recurrent or metastatic breast cancer: ESMO Clinical Practice Guidelines for diagnosis, treatment and follow-up. Annals of oncology: official journal of the European Society for Medical Oncology.

[37] Yang W (2011). Nuclear PKM2 regulates beta-catenin transactivation upon EGFR activation. Nature.

[38] Wang W, Luo YP (2015). MicroRNAs in breast cancer: oncogene and tumor suppressors with clinical potential. *Journal of Zhejiang University*. Science. B.

[39] van Schooneveld E (2015). Dysregulation of microRNAs in breast cancer and their potential role as prognostic and predictive biomarkers in patient management. Breast cancer research: BCR.

[40] Liu X (2015). MicroRNA-101 inhibits cell progression and increases paclitaxel sensitivity by suppressing MCL-1 expression in human triple-negative breast cancer. Oncotarget.

[41] Su CM (2016). miR-520h is crucial for DAPK2 regulation and breast cancer progression. Oncogene.

[42] Chen J (2011). Shikonin and its analogs inhibit cancer cell glycolysis by targeting tumor pyruvate kinase-M2. Oncogene.

[43] Li W, Liu J, Jackson K, Shi R, Zhao Y (2014). Sensitizing the therapeutic efficacy of taxol with shikonin in human breast cancer cells. PloS one.

[44] VanKlompenberg MK, Bedalov CO, Soto KF, Prosperi JR (2015). APC selectively mediates response to chemotherapeutic agents in breast cancer. BMC cancer.

[45] Martin EC (2012). Insulin-like growth factor-1 signaling regulates miRNA expression in MCF7 breast cancer cell line. PloS one.

[46] Park YS (2016). AKT-induced PKM2 phosphorylation signals for IGF-1-stimulated cancer cell growth. Oncotarget.

