# Supplementary material for: IGF-1-mediated PKM2/β-catenin/miR-152 regulatory circuit in breast cancer
Source: Sci Rep. 2017 Nov 21;7:15897. doi: 10.1038/s41598-017-15607-y (PMC5698474; doi:10.1038/s41598-017-15607-y)
Supplement: Supplementary file 1 — Supplemental materials [file 41598_2017_15607_MOESM1_ESM.doc]

**IGF-1-mediated PKM2/β-catenin/miR-152 regulatory circuit in breast cancer**

Yi-Yang Wen^1^, Wei-Tao Liu^1^, Hao-Ran Sun^1^, Xin Ge^1^, Zhu-Mei Shi^1,2^, Min Wang^1^, Wei Li^3^, Jian-Ying Zhang^4^, Ling-Zhi Liu^5^, Bing-Hua Jiang^1,5*^

1. State Key Laboratory of Reproductive Medicine, Key Laboratory of Human Functional Genomics of Jiangsu Province, Collaborative Innovation Center for Cancer Personalized Medicine, Jiangsu Key Laboratory of Cancer Biomarkers, Prevention, and Treatment, Cancer Center, and Department of Pathology, Nanjing Medical University, Nanjing, Jiangsu, China.

2. Department of Neurosurgery, The First Affiliated Hospital of Nanjing Medical University, Nanjing, China.

3. Department of Pathology, Affiliated Drum Tower Hospital of Nanjing University, Medical School, Nanjing, China.

4. Department of Biological Sciences & NIH-Sponsored Border Biomedical Research Center, The University of Texas at El Paso, El Paso, TX 79968, United States of America.

5. The Center for Molecular Carcinogenesis, Department of Pathology, Anatomy and Cell Biology, Thomas Jefferson University, Philadelphia, United States of America.

*Corresponding author: Bing-Hua Jiang, email: [binghjiang@yahoo.com](mailto:binghjiang@yahoo.com).

**Supplementary Figure S1**

**IGF-1 induces β-catenin and PKM2 nuclear accumulation.**

The subcellular localization of β-catenin and PKM2 in MCF7 and MDA-MB-231 cells treated with or without IGF-1 (24 hours) was determined by using Immunofluorescence assay with antibodies against β-catenin and PKM2, respectively. Scale bar: 10 μm.


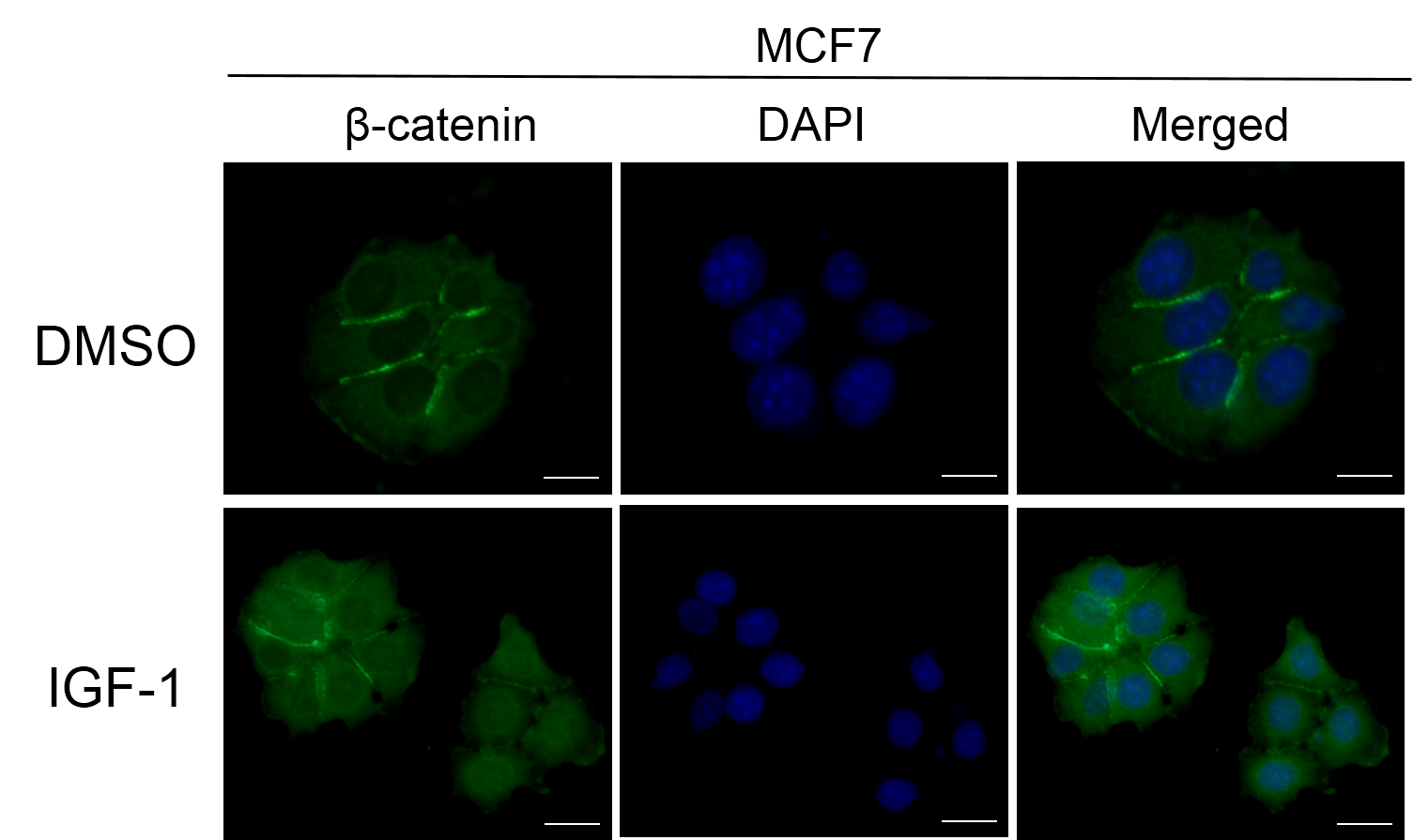

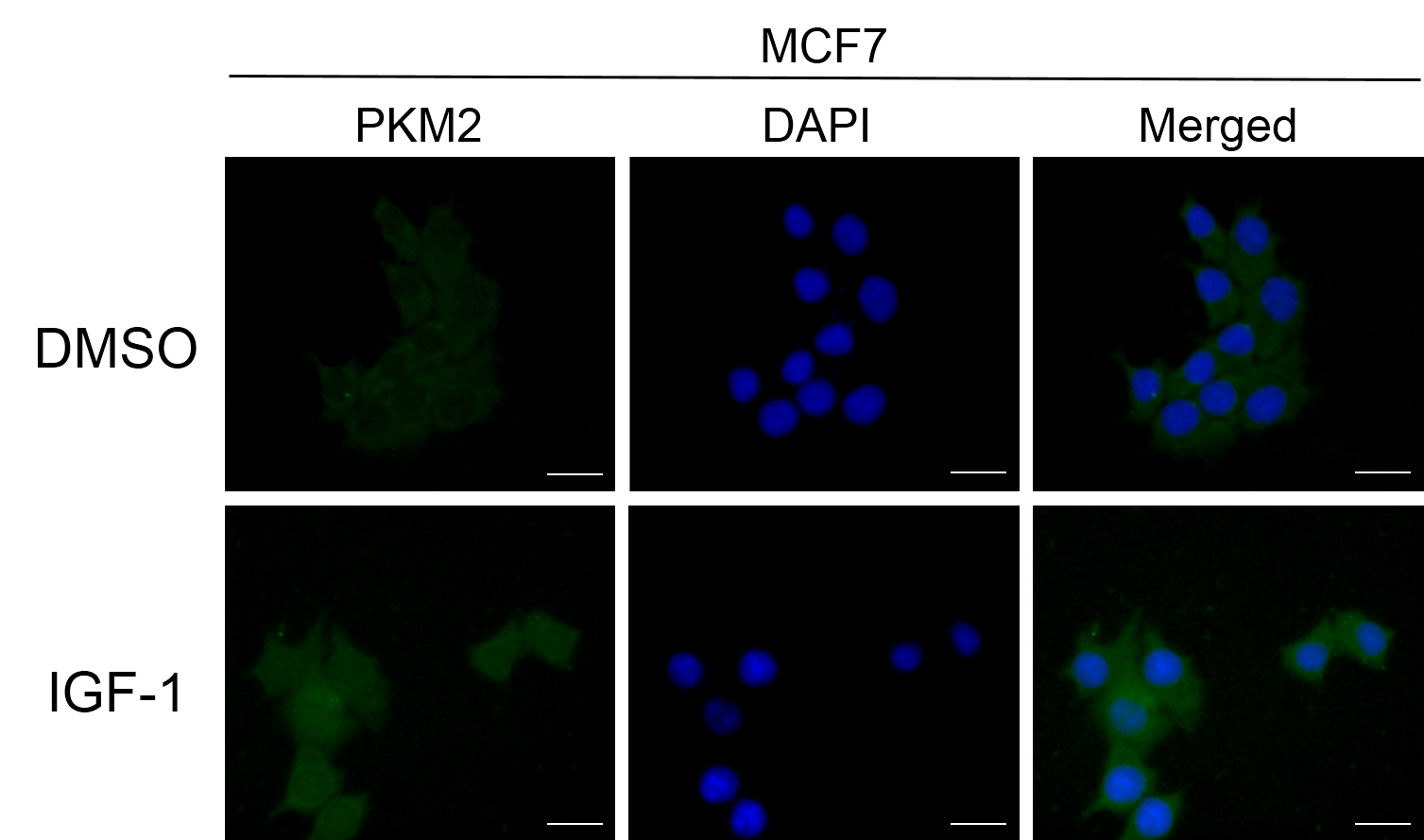


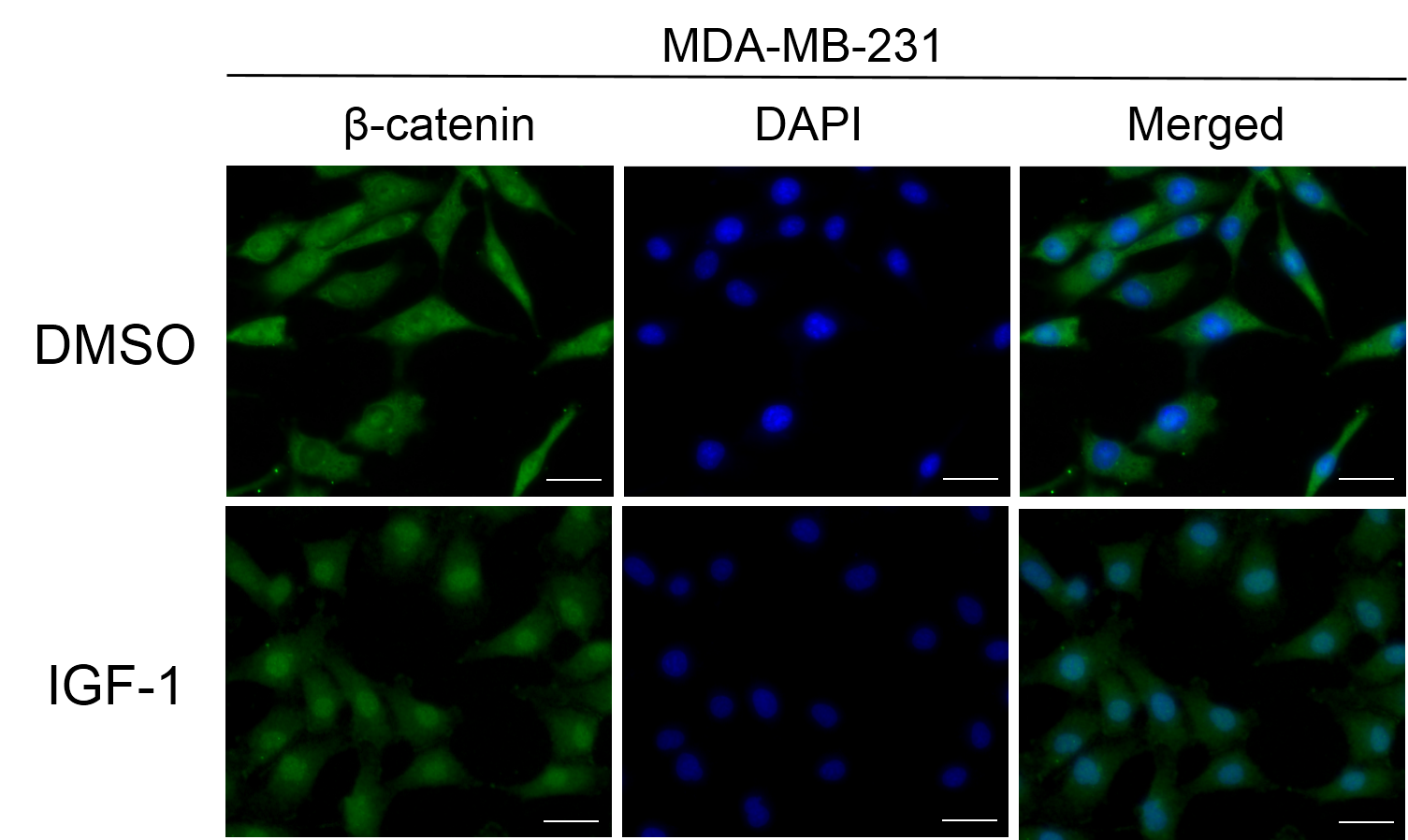

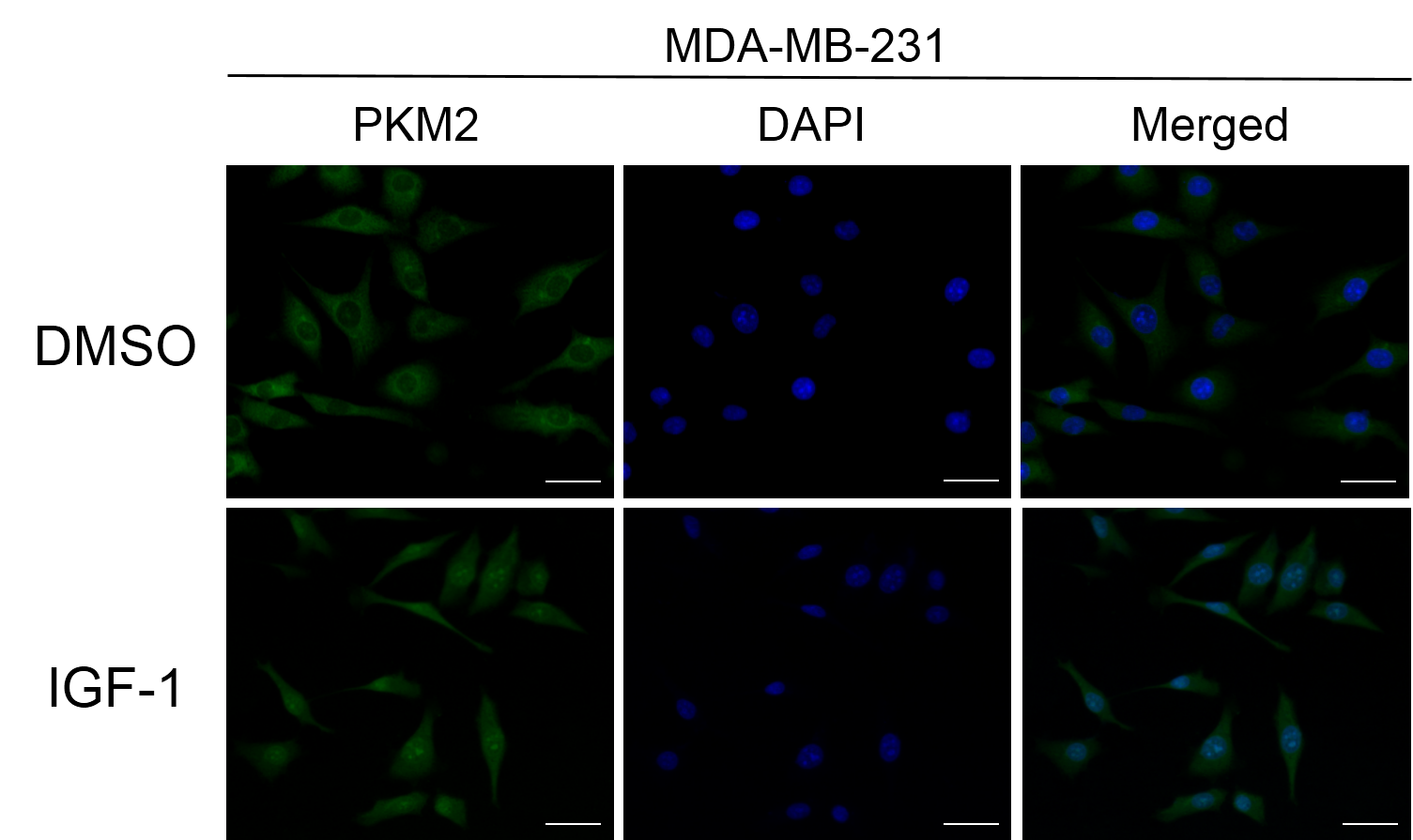


**Supplementary Figure S2**

**Knockdown of IGF-1R attenuates miR-152 expression level.**

The level of miR-152 was suppressed by sh-RNA-mediated knockdown of IGF-1R expression in MCF7 cells, but not in MDA-MB-231 cells; * indicates p<0.05 when compared to sh-RNA negative control.

**
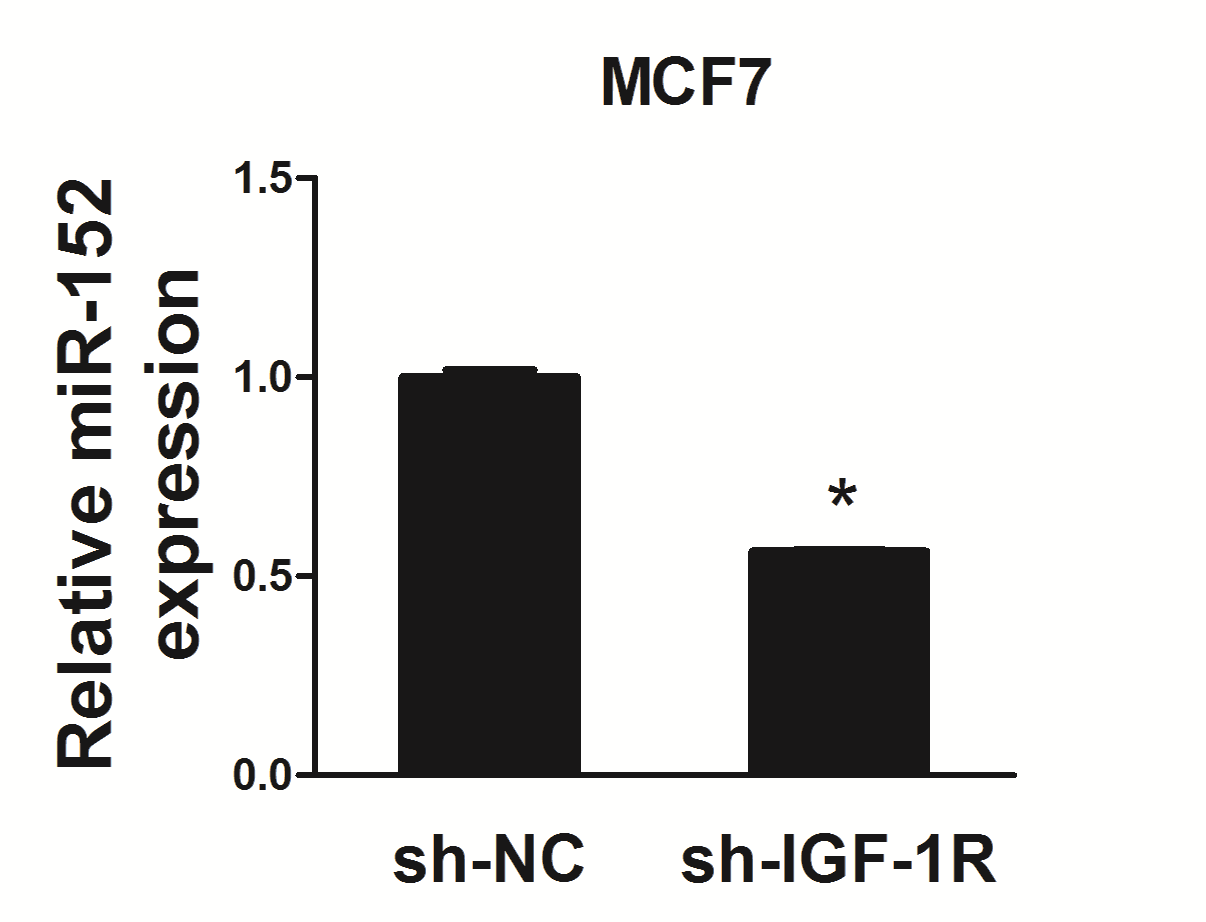

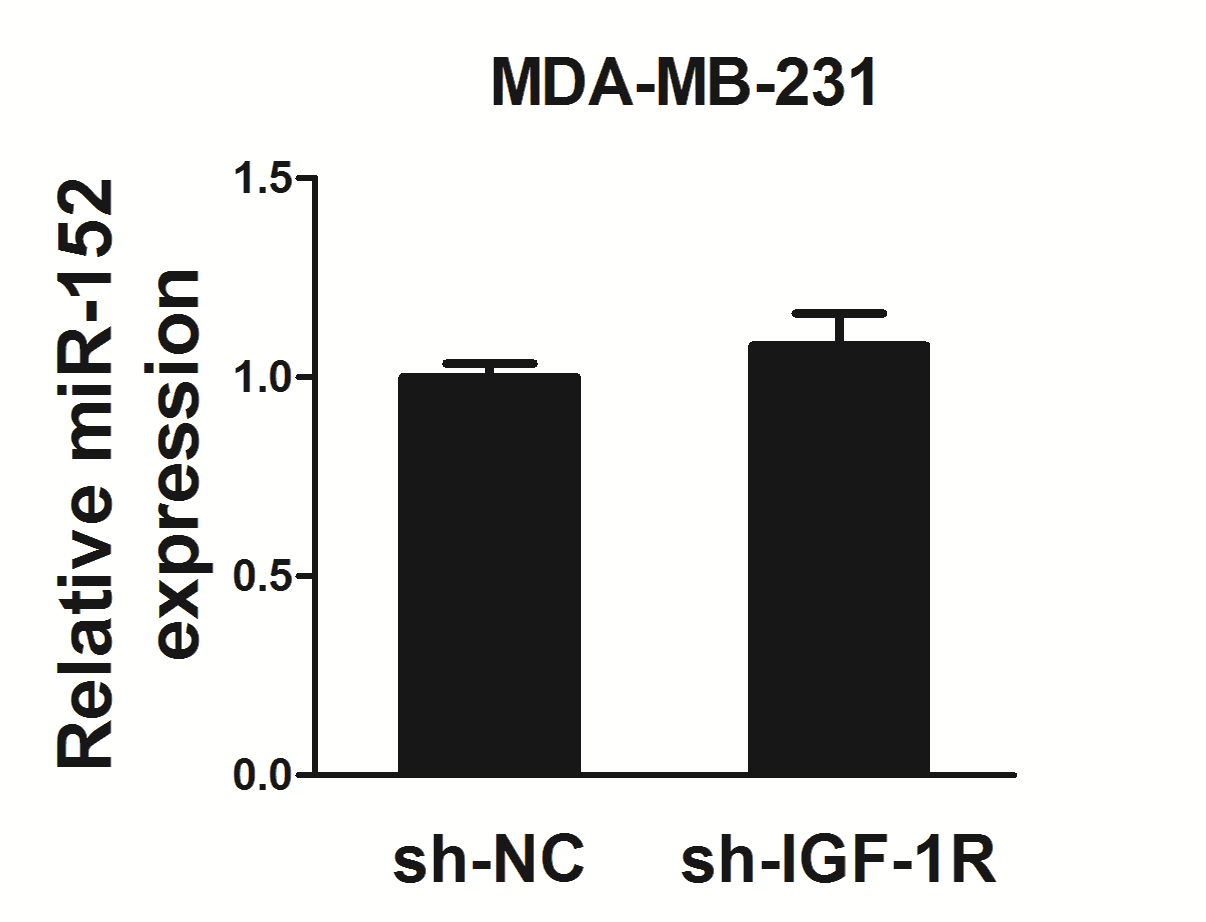
**

**Supplementary Table S1**

**Primers used in this study**

| **3'-UTR constructs primers** | |
| --- | --- |
| PKM2  (WT) | F: 5'- TCAGAGCTCCAACGCTTGTAGAACTCACTC -3'  R: 5'- CCGCTCGAGAAATGGAAGGTGGAGGG -3' |
| PKM2  (Mut) | F: 5'- TCAGAGCTCACTCTGGGCTGTAACGTGAGTCTGG -3'  R: 5'- CCGCTCGAGCCGCTCGAGAAATGGAAGGTGGAGGG -3 |
| β-catenin  (WT) | F: 5'- TCAGAGCTCTTTTGTTCTGGTCCTT-3'  R: 5'-CTGCTCGAGTAGATCCACCTGCTTAT-3' |
| β-catenin  (Mut) | F: 5'- AGAATATCTGTAATGGTACGGCCT-3'  R: 5'-CTGCTCGAGTAGATCCACCTGCTTAT-3' |
| **RT primers** | |
| U6 | 5'-AACGCTTCACGAATTTGCGT-3' |
| miR-152 | 5'GTCGTATCCAGTGCAGGGTCCGAGGTATTCGCACTGGATACGACCCAAGT-3' |
| **qPCR primer** |  |
| U6 | F: 5'- CTCGCTTCGGCAGCACA-3'  R: 5'- AACGCTTCACGAATTTGCGT -3' |
| miR-152 | F: 5'-GTGCAGGGTCCGAGGT-3'  R: 5'- TGACAGAACTTGGGTCGT-3' |
| PKM2 | F: 5'-TTGCAGCTATTCGAGGAACTCCG -3'  R: 5'-CACGATAATGCCCCACTGC-3' |
| β-catenin | F: 5'- CATCTACACAGTTTGATGCTGCT -3'  R: 5'- GCAGTTTTGTCAGTTCAGGGA-3' |
| β-actin | F: 5'-ATGGATGACGATATCGCTGCGC-3'  R: 5'- GCAGCACAGGGTGCTCCTCA -3' |
